# A novel spinal orthosis with embedded shape memory alloy for adolescent idiopathic scoliosis

**DOI:** 10.1186/s12938-026-01588-5

**Published:** 2026-05-20

**Authors:** Peikun Zhu, Chunxiao Lu, Xinjing Wu, Jinzhu Bai

**Affiliations:** 1 School of Rehabilitation Medicine, Shandong University of Traditional Chinese Medicine, Jinan, 250355 Shandong China; 2 Department of Spine and Spinal Cord Surgery, Beijing Boai Hospital, China Rehabilitation Research Center, Beijing, 100068 China; 3 School of Rehabilitation Medicine, Capital Medical University, Beijing, 100069 China

**Keywords:** Adolescent idiopathic scoliosis, Spine orthosis, Brace, Shape memory alloy, Clinical treatment

## Abstract

Bracing is the most effective measure for the treatment of mild-to-moderate adolescent idiopathic scoliosis (AIS). However, the balance between three-dimensional correction of scoliosis and wear comfort is still not ideal. To design a novel bracing with embedded shape memory alloy for the treatment of AIS that can improve patients’ comfort and provide sustained and effective three-dimensional correction force, as well as to preliminarily verify its clinical efficacy. A Ni‒Ti shape memory alloy material was constructed with 20 ℃ martensite and 0 ℃ austenite. On the basis of the traditional rigid thoracolumbosacral scoliosis braces, when considering the three-dimensional correction of scoliosis and the wear comfort of the patient, a novel spinal orthosis with embedded shape memory alloy was designed and manufactured, and the performance of the novel brace was tested. Ten patients with mild-to-moderate scoliosis were included to evaluate clinical efficacy. The bending test and minimum surface temperature of the embedded shape memory alloys met the deformation requirements for the novel brace. The thoracic kyphosis and lumbar lordosis angle were larger and the interface pressure was lower in the novel brace group than in the traditional brace group (*P* < 0.05). The SRS-22 assessment with the novel brace was better than with the traditional brace (*P* < 0.05). The novel brace provided a better three-dimensional correction effect to adolescent idiopathic scoliosis patients and also increased wearing comfort than traditional orthoses, which can improve compliance of patients.

## Introduction

Scoliosis is radiologically defined as a lateral curvature of more than 10° with rotation of the spine [1]. Adolescent idiopathic scoliosis (AIS) is the most common type of scoliosis. According to the Scoliosis Research Society (SRS), the prevalence of AIS in the general population is approximately 2–3%, and 10% of patients require treatment. Ultimately, 0.1–0.3% of patients require surgery [2].

For moderate scoliosis patients with Cobb angles between 20° and 45° or mild scoliosis patients with significant progression during a short period of time, the conventional treatment method is to wear scoliosis braces [3]. The Bracing in Adolescent Idiopathic Scoliosis Trial (BrAIST) study demonstrated the effectiveness of bracing for the treatment of AIS, which has been recognized worldwide [4]. According to the data of the SOSORT Idiopathic Scoliosis Rehabilitation guidelines, bracing is the most effective measure for the treatment of mild-to-moderate AIS, and the level of evidence-based medicine is the highest [5].

The therapeutic effect of braces is related to many factors, such as wearing time, patient condition, and type of brace. Due to its own characteristics, the effect of flexible braces on the treatment of scoliosis is limited [6]. At present, rigid braces are still widely used in the clinical setting. However, traditional rigid braces have common shortcomings, such as difficult adjustment, stiffness, irritation of the skin, and limited muscle activity. We reviewed the literature and referred to the feedback from patients who had previously worn traditional orthoses. Those problems will affect patient compliance, which will lead to a reduction in treatment efficacy [7]. Currently, most braces are focused on the coronal lateral curvature of the spine, and three-dimensional correction (i.e., correction of lateral bending, axial rotation, rib protrusion and spinal kyphosis occurring simultaneously) is still not ideal [8]. This may be related to the defects of traditional orthoses, that is, the traditional rigid devices mainly adopt the two-dimensional three-point force principle while ignoring the three-dimensional nature of scoliosis. Although some orthoses are equipped with corrective forces applied from multiple directions, from the perspective of any single corrective dimension, the corrective force is still a single-directional rigid static compression. Moreover, rigid brace materials may deteriorate and slack during long-term wear, thus leading to the loss of orthopedic force [9]. For a long period of time, scholars have attempted to improve bracing methods, such as via finite element technology (FEM) or computer-aided design technology, in order to overcome certain shortcomings [10–12]; however, the improvement of the treatment effect, especially for the three-dimensional correction of lateral curves, is still not obvious [13–15].

Based on the shortcomings of traditional rigid braces, the choice of materials may be helpful for improving the efficacy of scoliosis orthosis. Shape memory alloy (SMA) is an intelligent metal with a shape memory effect [16]. The shape of the alloy changes with temperature, which is essentially the mutual transformation of different structures and states of the alloy. When the temperature decreases to a specific point, the austenite transforms into twin martensite, and the alloy undergoes plastic deformation [17]. After the twin martensite is loaded and molded, it is redirected to nontwin martensite. As the temperature increases, the nontwin martensite transforms to relatively rigid austenite, which demonstrates that SMAs return to their preset shape [18]. At present, mature SMAs mainly include Ni–Ti-based alloys, copper-based alloys and iron-based alloys, among which the Ni–Ti alloy has the best performance. Cheung [19] used an SMA rod to surgically treat AIS patients and conducted a randomized controlled experiment to confirm its safety and effectiveness. Cheung [19] performed surgical treatment on adolescent idiopathic scoliosis patients using a shape memory alloy rod, and verified its safety and effectiveness through a randomized controlled experiment. Chan [20] utilized the high elasticity characteristic of shape memory alloy and applied it to the resin pillars of the flexible corrective device, thereby achieving better thoracic spine correction results. However, the risk of surgical intervention is significantly higher, while the corrective effect of the soft corrective device has not yet been confirmed in clinical practice.

To address the shortcomings of the traditional rigid brace, on the basis of the principle of three-dimensional correction and referring to related research on SMAs, this study strived to combine an Ni–Ti SMA with a traditional thoracolumbosacral orthosis to design and develop a spinal orthosis with embedded shape memory alloy and preliminarily applied it in clinical practice to evaluate its efficacy. Because of the shape memory effect, the brace could be individually adapted at low temperature. After the temperature increased, the brace could be restored to the preset shape. Multiple orthopedic pads acted simultaneously to generate orthopedic force, which aimed to improve the wearing comfort of patients and provided patients with sustained and effective three-dimensional corrective force, thus providing a novel brace for the three-dimensional correction of AIS. We attempted to design a new type of orthosis, which can effectively enhance the three-dimensional correction effect and increase patients’ compliance with wearing it.

## Results

### The brace sample

**Fig. 1:**
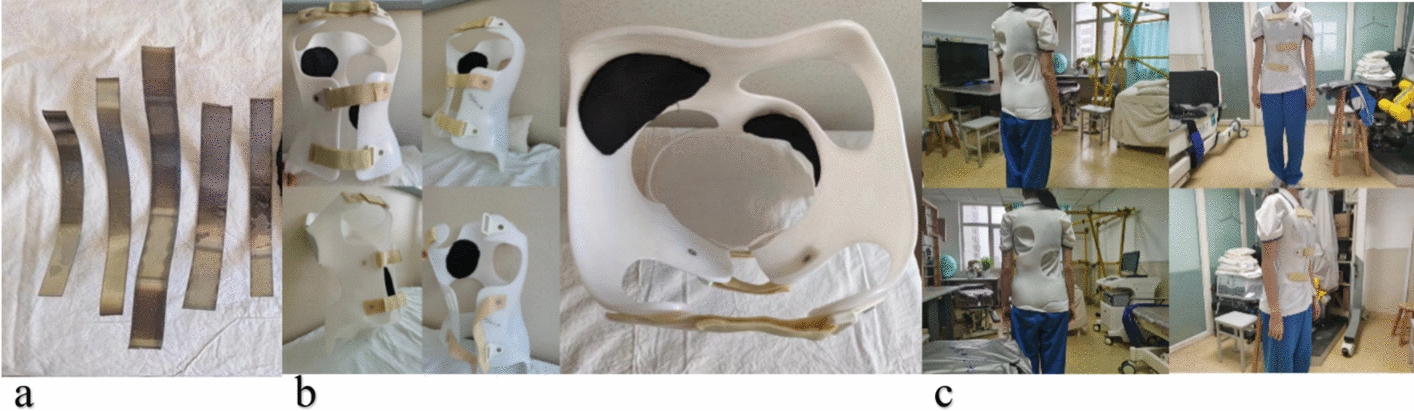
Finished brace and patient fitting. Preset shape of the shape memory alloy orthopedic pad (**a**); specialized brace shell with an embedded shape memory alloy orthopedic pad and an elastic knitted cover (**b**); schematic diagram of the patient wearing the device (**c**)

We completed the manufacturing of the shape memory alloy orthopedic pad and conducted patient fitting tests. The complete orthosis consists of a shell and a shape memory alloy orthopedic pad wrapped by an elastic knitted cover. The brace sample and patient wear are shown in Fig. 1. There was no discomfort reported while wearing the device.

### Performance test

#### Bending test

For the SMA pieces of the three specifications, the compression resistance increased with increasing width and decreasing length of the SMA, and the 20 mm width had the best rigidity and strongest compression resistance. The detailed experimental results are shown in Table 1.

**Table 1:** Compression resistance of the SMA (*N*)

Length					
Depth	40 cm	30 cm	20 cm	10 cm	width
5 mm	1.21 ± 0.10	1.87 ± 0.06	3.24 ± 0.15	6.23 ± 0.09	10 mm
10 mm	2.63 ± 0.07	3.20 ± 0.09	6.42 ± 0.12	12.28 ± 0.08	
15 mm	4.00 ± 0.19	5.15 ± 0.13	10.2 ± 0.21	17.69 ± 0.13	
20 mm	5.25 ± 0.16	6.97 ± 0.09	14.36 ± 0.20	23.63 ± 0.52	
5 mm	2.13 ± 0.14	3.45 ± 0.11	9.41 ± 0.23	14.34 ± 0.13	15 mm
10 mm	3.56 ± 0.16	5.21 ± 0.10	16.67 ± 0.16	26.51 ± 0.07	
15 mm	6.70 ± 0.09	7.87 ± 0.24	24.55 ± 0.17	39.62 ± 0.18	
20 mm	9.28 ± 0.14	10.20 ± 0.38	36.51 ± 0.23	51.23 ± 0.08	
5 mm	3.64 ± 0.29	5.67 ± 0.10	18.27 ± 0.07	30.35 ± 0.14	20 mm
10 mm	6.72 ± 0.03	9.83 ± 0.10	29.35 ± 0.11	44.75 ± 0.14	
15 mm	8.32 ± 0.13	11.88 ± 0.12	41.34 ± 0.11	62.66 ± 0.24	
20 mm	11.27 ± 0.51	16.90 ± 0.29	56.74 ± 0.11	80.25 ± 0.22	

#### Brace surface temperature

The room temperature was generally approximately 25 °C, the temperature of the austenite phase transition of the SMA was 20 °C; and the surface temperature of the brace in different seasons and different states was more than 20 °C. Details are shown in Table 2.

**Table 2:** Temperature of the brace surface (°C)

Season		Rehabilitation training	
		Yes	No
Spring	Indoor	30.77 ± 0.76	29.39 ± 0.59
	Outdoor	29.34 ± 0.51	28.38 ± 0.66
Summer	Indoor	32.29 ± 0.59	29.52 ± 0.73
	Outdoor	33.81 ± 0.77	32.33 ± 0.64
Autumn	Indoor	30.53 ± 0.62	29.22 ± 0.47
	Outdoor	29.37 ± 0.45	27.79 ± 0.41
Winter	Indoor	29.65 ± 0.55	27.71 ± 0.47
	Outdoor	28.44 ± 0.40	25.51 ± 0.48

#### The temperature of the shape memory alloy

**Fig. 2:**
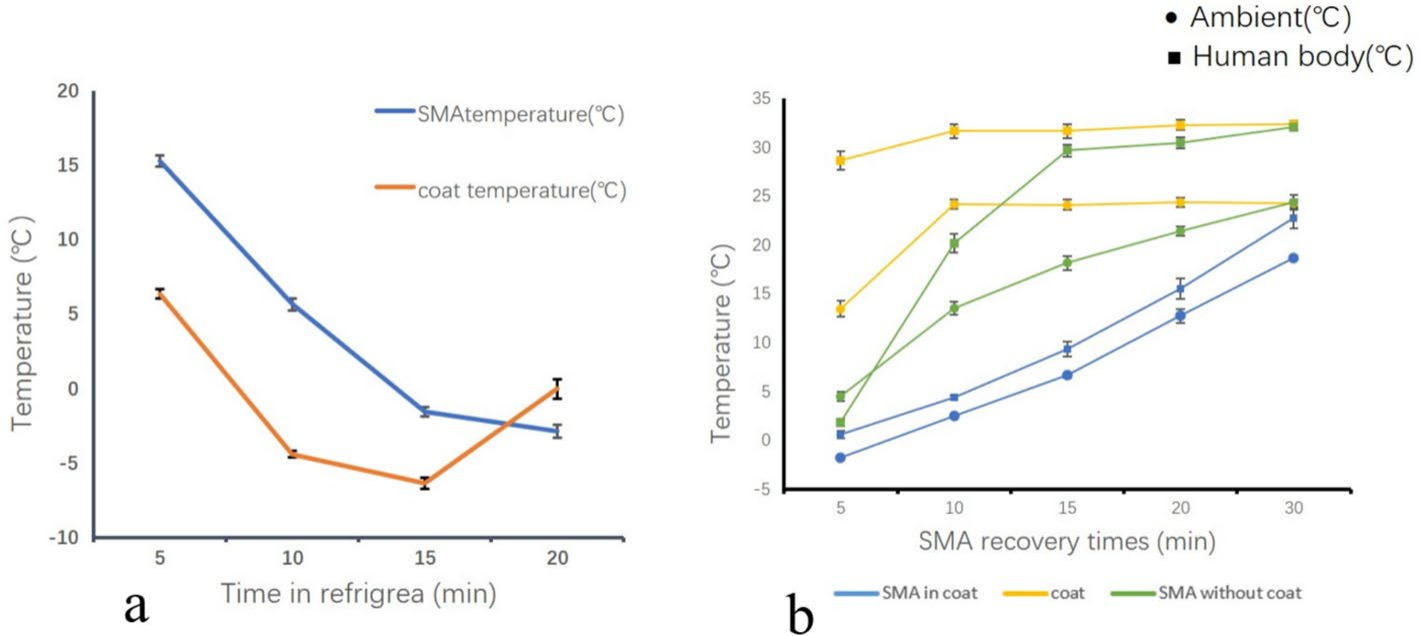
Temperature changes of the SMA in the refrigerator (**a**) and the SMA recovery at different times (**b**)

The martensite transformation temperature of the SMA was 0 °C, and the temperature of the orthopedic pieces reached -1.56 ± 0.33 °C in a freezer for 15 min, which met the molding requirements. The average temperature reached 15.50 ± 1.05 °C at 20 min and 22.68 ± 0.96 °C at 30 min after the orthosis was removed from the low-temperature environment and applied to the human body. At ambient temperature, the coat surface reached 13.44 ± 0.80 °C after 5 min and 24.14 ± 0.46 °C after 10 min. The details are shown in Tables 3 and 4. The temperature changes of the SMA in different environments are shown in Fig. 2.

**Table 3:** Temperature of the SMA in the refrigerator at different times (°C)

Time in refrigerator	SMA temperature	Coat temperature
5 min	15.32 ± 0.36	6.40 ± 0.32
10 min	5.66 ± 0.39	−4.40 ± 0.20
15 min	−1.56 ± 0.33	−6.38 ± 0.38
20 min	−2.88 ± 0.43	−7.40 ± 0.65

**Table 4:** Temperature of the SMA recovery at different times (°C)

		SMA in coat	Coat	SMA without coat
Ambient	5 min	−1.76 ± 0.13	13.44 ± 0.80	4.48 ± 0.44
	10 min	2.48 ± 0.18	24.14 ± 0.46	13.50 ± 0.64
	15 min	6.62 ± 0.22	24.10 ± 0.56	18.14 ± 0.72
	20 min	12.72 ± 0.72	24.32 ± 0.45	21.40 ± 0.50
	30 min	18.68 ± 0.15	24.23 ± 0.40	24.32 ± 0.74
Human body	5 min	0.62 ± 0.38	28.60 ± 0.94	1.82 ± 0.39
	10 min	4.38 ± 0.23	31.62 ± 0.70	20.16 ± 0.95
	15 min	9.32 ± 0.74	31.64 ± 0.73	29.66 ± 0.62
	20 min	15.50 ± 1.05	32.24 ± 0.52	30.40 ± 0.58
	30 min	22.68 ± 0.96	32.30 ± 0.42	32.06 ± 0.44

#### The deformation time of the orthopedic pad

The average recovery time of the orthopedic pad was 33.62 ± 1.52 min at ambient temperature and 24.12 ± 1.08 min when applied to the human body (Table 5).

**Table 5:** Deformation time of the orthopedic pad (min)

	Ambient	Human body
Time	33.62 ± 1.52	24.12 ± 1.08

#### Clinical evaluation

A total of 10 patients were included in this clinical study; no patients dropped out, and no serious adverse events occurred.

#### Baseline

The clinical and demographic baseline characteristics of the patients are shown in Table 6. There were no differences between the two groups (*P* > 0.05).

**Table 6:** Baseline characteristics of patients

	Novel brace group	Control group	*p* value
Age (y)	11.60 ± 1.52	12.40 ± 2.30	0.43
Height (cm)	152.20 ± 6.38	153.00 ± 8.80	0.87
Weight (kg)	45.42 ± 4.41	46.00 ± 7. 28	0.88
Sex (F/M)	4/1	3/2	0.39
Lenke I/V	3/2	4/1	0.50
Risser sign (0/1/2/3/4/5)	0/2/1/1/1/0	0/0/1/2/2/0	0.19
Cobb Angle (°)	29.40 ± 7.70	30.80 ± 6.42	0.66
AVR (0/1/2/3/4)	0/1/3/1/0	0/1/4/0/0	0.61
TKA (°)	28.4 ± 6.54	27.80 ± 3.11	0.43
LLA (°)	38.2 ± 3.63	36.4 ± 2.07	0.88

*TKA* Thoracic Kyphosis Angle, *LLA* Lumbar Lordosis Angle, *AVR* Apical Vertebra Rotation

#### Radiographic examination

After 6 months of intervention, the Cobb angle decreased in both groups, but there was no significant difference between the two groups (*P* > 0.05). The thoracic kyphosis angle in the novel brace group did not significantly improve but was greater than that in the control group (*P* < 0.05). The lumbar lordosis angle significantly improved (*P* < 0.05) and was greater than that of the control group (*P* < 0.01). There was no significant difference in the improvement in the vertebral rotation angle between the two groups. The details are shown in Table 7.

**Table 7:** Assessments before and after treatment

Indicators	Groups	Before treatment	End of treatment	*p* value (within-group)	Difference	*p* value (between-group)
Cobb angle (°)	Novel	28.80 ± 7.46	23.20 ± 7.79	0.03	5.60 ± 3.78	0.89
	Control	30.80 ± 6.42	24.80 ± 3.27	0.07	6.00 ± 5.52	
TKA (°)	Novel	28.4 ± 6.54	29.4 ± 3.85	0.37	−3.20 ± 7.12	0.047
	Control	27.80 ± 3.11	24.00 ± 1.87	0.02	3.80 ± 2.28	
LLA (°)	Novel	38.2 ± 3.63	43.6 ± 4.67	0.02	−5.00 ± 1.58	0.008
	Control	36.4 ± 2.07	34.4 ± 2.30	0.61	2.00 ± 1.73	
AVR (0/1/2/3)	Novel	0/1/3/1	1/3/1/0	/	/	0.14
	Control	0/1/4/0	1/1/2/1	/	/	

*TKA* Thoracic Kyphosis Angle, *LLA* Lumbar Lordosis Angle, *AVR* Apical Vertebra Rotation

#### SRS-22 and QUEST

No difference was observed between the groups in the scores on the four domains of the SRS-22 except for satisfaction. The novel brace improved the self-image and mental health scores after treatment. The QUEST in the novel brace group was greater than that in the control group (Table 8).

**Table 8:** Comparison of the questionnaire

	Before treatment		End of treatment		*p* value (between-group)
	Novel	Control	Novel	Control	
SRS-22					
Function	24.20 ± 0.84	23.4 ± 0.89	24.8 ± 0.45	23.8 ± 0.84	0.75
Pain	23.60 ± 1.14	22.8 ± 0.84	24.00 ± 1.41	22.80 ± 0.45	0.50
Mental health	17.40 ± 2.07	16.4 ± 1.82	19.80 ± 3.27*	17.00 ± 1.58	0.14
Self-image	17.60 ± 0.55	16.40 ± 1.14	20.00 ± 2.35*	17.40 ± 1.14	0.23
Satisfaction	/	/	9.60 ± 0.55	7.40 ± 1.14	0.01
Total	82.80 ± 3.11	79.00 ± 1.58	88.60 ± 7.16*	81.00 ± 2.35	0.25
Quest	/	/	59.20 ± 0.85	55.40 ± 2.30	0.01

^*^indicates comparisons before and after treatment, P < 0.05; there was no significant difference in scores between the two groups of patients at the initial consultation

#### Interface pressure

The measurement of pressure is taken when the patient is breathing normally and the data are recorded at the end of inhalation. There was a significant difference between the two groups (Table 9), and the interface pressure of the traditional brace was significantly greater than that of the novel brace (*P* = 0.008).

**Table 9:** Comparison of the interface pressures (*Kpa*)

	Novel group	Control group	*p* value
Pre-deformation	12.50 ± 6.22	/	
Post-deformation	81.14 ± 13.95	139.09 ± 11.01	0.008

#### Illustrative case

**Fig. 3:**
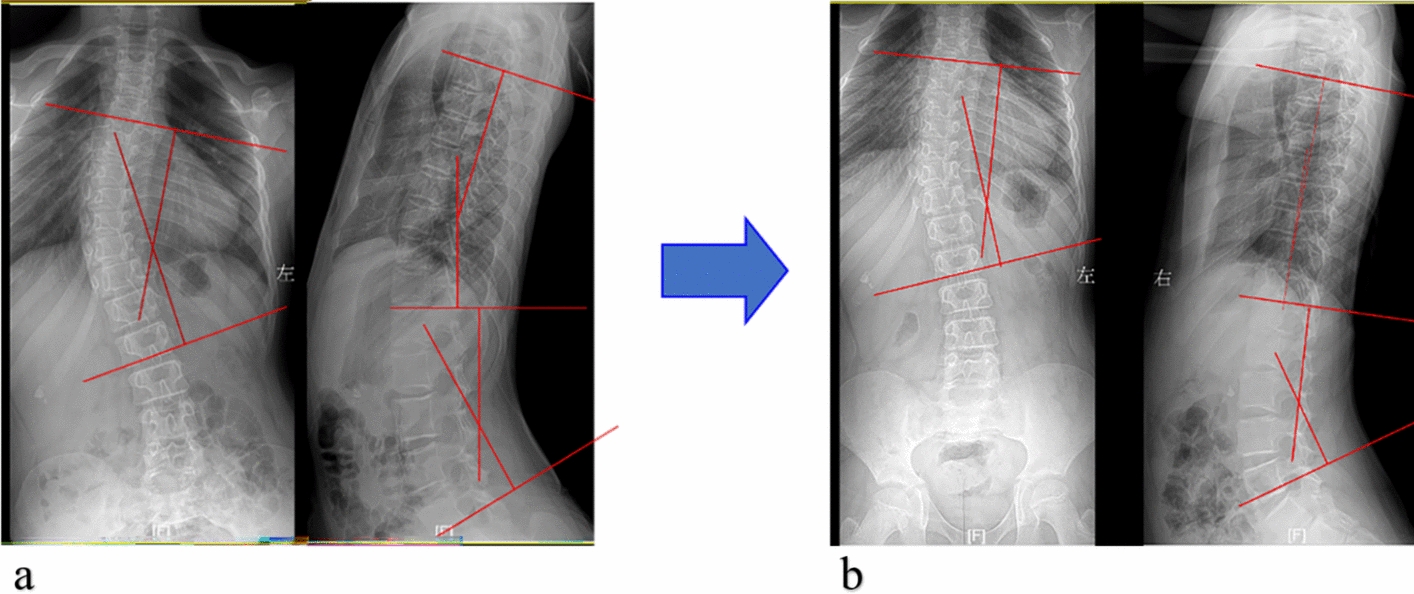
X-rays of patients before (**a**) and after treatment (**b**)

The patient was a 12-year-old male thoracic unicurve individual with a height of 146 cm and a weight of 43 kg. The initial Cobb angle was 30°, the thoracic kyphosis angle was -18°, the lumbar anterior kyphosis angle was 29°, and the vertebral body rotation angle was 2°. After 6 months of treatment with the novel brace, the Cobb angle improved by 12°, the thoracic kyphosis angle improved by 15°, the lumbar lordosis angle improved by 3°, and the vertebral rotation angle improved by 1° (Fig. 3).

## Discussion

In order to balance three-dimensional correction of scoliosis and wear comfort, a novel spinal orthosis with embedded shape memory alloy was designed in this study. The novel brace was tested and clinically evaluated. The results showed that it has a better three-dimensional correction effect for adolescent idiopathic scoliosis patients and higher wear comfort than traditional orthoses, which may improve compliance of patients. Further research on patients’ compliance, including quantitative metrics, such as average wearing time, will be supplemented and refined in our subsequent studies.

### Performance test of brace

#### Bending test

The purpose of this experiment was to test the compressive performance of an SMA piece with different specifications and to select the specifications suitable for orthopedic requirement. Ultimately, an SMA with a width of 20 mm was used to produce an orthopedic pad. This study targeted adolescents with idiopathic scoliosis. According to Cobetoo et al. [23], the pressure threshold that the trunk can withstand when wearing a brace is 35 kPa. When considering both corrective effects and patient tolerance, the optimal area for correction is between 64 cm^2^ and 225 cm^2^, with side lengths ranging from 8 to 15 cm, and the corrective force should not exceed 100 N [24]. Previous studies have indicated that the corrective force of orthoses generally ranges from 20 to 60 N [8, 11], with the greatest corrective force occurring at the thoracic vertebrae. The orthopedic pad is composed of multiple SMA strips arranged in combination to collectively withstand pressure. Excluding active correction forces provided by muscles and respiration factors, theoretically, the orthopedic pad should be able to deform smoothly to provide sufficient corrective force.

#### The interface temperature of the brace

The purpose of this experiment was to test the temperature when wearing an orthosis for different durations and at different states to determine whether the temperature required for the phase transition of SMA can be reached. The results indicated that during the use of a novel brace, the temperature can reach the phase transition temperature of the SMA, and the orthopedic pad can completely deform.

#### The temperature of the shape memory alloy

The purpose of this experiment was to determine the duration required for SMA to reach its deformation temperature when frozen in a refrigerator, as well as the time it takes to reach different temperatures after deformation at worn or ambient temperatures. The results indicated that the SMA reached the required plasticity within 20 min in the freezer compartment. Upon removal from the refrigerator, at ambient temperature, the average surface temperature of the SMA reached 13.44 ± 0.80 °C at 5 min and 24.14 ± 0.46 °C at 10 min, thus causing no significant discomfort when worn by individuals. When applied to a human body, it takes an average of 20–30 min to fully reach the temperature required for restoring its preset shape. In theory, after shaping, SMA orthopedic pads gradually return to their preset shape within 30 min, and corrective force is applied to patients.

#### The deformation time of the orthopedic pad

The aim of this experiment was to ascertain the time needed for an SMA orthopedic pad to fully return to its preset shape after deformation, thus ensuring that patients can undergo successful shaping and donning. Once the orthopedic pad was shaped and donned, it fully recovered to its preset shape in an average of 24.12 ± 1.08 min. By slowly applying corrective force to the patient’s torso, the patient gradually adapts to the pressure, thus reducing discomfort while donning and enhancing compliance to wearing the novel brace.

#### Preliminary clinical application

This study preliminarily validated the clinical efficacy of the novel spinal orthosis with embedded SMA, analyzed 10 AIS patients, and compared the outcomes of patients before and after treatment with the traditional brace. Upon evaluation of the coronal plane effects, after 6 months of clinical treatment, the novel brace significantly reduced the patients’ coronal plane Cobb angle, with an average improvement of 5.60 ± 3.78° (*P* = 0.03). It was basically the same as that of the traditional brace group (5.60 ± 3.78° vs. 6.00 ± 5.52°, respectively), with no significant difference (*P* = 0.89). The results indicated that the correction effect of the novel brace in the coronal plane was at least not inferior to that of traditional braces. This may be because the traditional brace itself has already achieved good results in the correction of the coronal plane, while the new brace pays more attention to the three-dimensional changes of the spine, only improving the way and direction of orthopedic force, without fundamentally changing the biomechanics of the brace.

The novel brace tended to improve the sagittal spinal curvature after treatment, with a mean increase in thoracic kyphosis of 3.2 ± 7.12°, although this difference was not statistically significant (*P* = 0.37). Lumbar lordosis increased by a mean of 5.00 ± 1.58° (*P* = 0.02) after treatment. Compared with the traditional brace, the novel brace demonstrated significantly greater improvement in the sagittal curvature of the spine in patients with AIS. This effect may be attributed to the fact that the correction force is applied in a more proximal location, which was more conducive to increasing the lumbar lordosis angle. Previous studies [25–27] have demonstrated that thoracic kyphosis and lumbar lordosis interact with each other. The brace was designed to expand the posterior space of the non-orthopedic region of the thoracic spine, thereby reducing the restriction on the thoracic vertebrae, which resulted in a slight restoration of thoracic kyphosis. Furthermore, the novel brace was designed to facilitate active correction, which correspondingly encouraged the avoidance effect in patients, thus guiding the spine to be displaced to the healthy side. Previous studies [28–30] have indicated that traditional brace treatment was more likely to cause flat back deformity in patients. The results of this study suggested that patients in the traditional brace group had an average reduction in thoracic kyphosis of 3.80 ± 2.28° after treatment (*P* = 0.02). In contrast, the novel brace did not significantly increase the angle of thoracic kyphosis but at least maintained the original curvature, and a trend toward flat back deformity was not observed. This result may also be related to the size of the sample.

There was no significant difference in the improvement of apical vertebral rotation (AVR) between the two groups at 6 months after treatment. This effect was likely due to the relatively small sample size of this study. However, there was a tendency for the AVR of patients with the novel brace to decrease, indicating that the novel brace may be more advantageous for correcting the axial rotation of the spine. This may be attributed to the fact that the novel brace conformed to the patient’s body and provided a greater derotation in the gradual dynamic process of deformation of the orthopedic pad. In contrast, traditional braces exerted a correction force by directly pushing the lateral thorax through a transverse force, which restricted its further development.

The pressure measurement at the orthosis interface indicated that the pressure on the skin of patients in the novel brace group was significantly less than that of the traditional brace group. This difference was statistically significant. The mean pressure when the brace was in situ was 12.50 ± 6.22 kPa, indicating that when the SMA did not deform, the brace fitted to the patient’s body, and the patient did not feel obvious pressure, which was helpful to reduce the anxiety and discomfort of the patient when initially wearing the brace, and enhance the patient’s willingness to wear. The average pressure experienced by patients wearing the novel brace was 81.14 ± 13.95 kPa after SMA deformation. Average interface pressure in the traditional control group was 139.09 ± 11.01 kPa, which was similar to that observed by Fuss (14–124 kPa) [31]. The novel brace group exhibited a significantly lower pressure than that of the control group. This difference was statistically significant (*P* = 0.008). The possible reason was that the SMA pad with a good fit to the trunk distributed the local orthopedic stress, thereby reducing the pressure on the patient’s deformity site. The magnitude of the interface pressure may affect the patient’s wearing compliance. The pressure-adjustable orthosis designed by Lin et al. [32] was worn 1.1 h longer per day than conventional braces. In this study, the interface pressure of the novel brace was significantly lower than that of the traditional brace, which may improve the wearing compliance of patients and thus enhance the orthopedic effect.

The SRS-22 questionnaire showed that the novel brace had higher satisfaction than the traditional brace. Functional and pain scores in both groups did not change significantly before and after treatment, and there were no significant differences between the two groups. This may be due to the fact that the subjects of this study were patients with mild-to-moderate scoliosis, with no obvious manifestations of pain and dysfunction. However, the novel brace could improve patients’ mental health and self-image perception. There were significant differences in QUEST scores between the two groups (*P* = 0.01), which indicated that patients had higher satisfaction with the novel brace.

#### The novel brace compared with the traditional rigid brace

In the novel SMA spinal orthosis designed in this study, the brace shell itself did not exert correction force, which could reduce the discomfort of wearing, improve the willingness to wear and increase the compliance of patients. At present, the active correction ability of traditional orthosis needs to be improved, while the novel brace pays more attention to active correction, and guides the spine to return to the normal physiological curve through the breathing movement of the human body and the avoidance effect of the muscles. Unlike traditional rigid braces that exert direct lateral compression on the thoracic cavity, the design of this novel type of brace is different. In low-temperature environments, the orthotic pads are custom-shaped according to the patient's anatomical structure. Under the influence of body heat, multiple orthotic pads gradually release corrective forces in multiple directions during the dynamic deformation process. This enables the patient to actively adjust their posture by breathing and muscle contraction into the space reserved by the brace. Due to the fact that the brace itself is in close contact with the patient's body, it can provide a larger derotation during the avoidance process, jointly guiding the spine to return to its normal structure. In terms of performance, the novel brace could achieve the desired effect, and the shape memory alloy could complete the deformation in a certain time and provide sufficient correction force. In terms of clinical efficacy, compared with the traditional rigid thoracolumbosacral brace, the novel brace could provide better three-dimensional orthopedic effects, improve the coronal, sagittal and cross-sectional deformities of patients to a certain extent, and reduce the incidence of flat back deformities. The assessment scale showed that the novel brace had a better clinical effect, and was helpful to improve the patient’s self-perception and reduce the patient’s anxiety. In terms of interfacial pressure, the novel brace generated less local pressure on the patient, which could reduce discomfort. The superelastic effect of shape memory alloys may also be one of the reasons for the improved comfort of braces. As the tissue viscoplasticity of the fasciae, prolonged pressure causes fiber creep; long-term repetitive loading may result in partial irreversible permanent deformation of the fascia, leading to problems, such as pain, stiffness and restricted movement. Prolonged use of traditional braces subjects patients to rigid, concentrated and sustained high pressure, which may cause permanent plastic changes in the fascia. In contrast, shape memory alloys possess superelasticity, allowing them to conform to the contours of the torso while distributing pressure evenly across the surface, thereby preventing localized stress concentrations. The orthopedic force is always stable and soft during the patient’s breathing, turning and other activities, and can return to its original shape almost completely, ensuring that no new stress points develop during long-term use.

In conclusion, the novel spinal orthosis with embedded SMA has clinical significance.

## Limitations

This study was a preliminary investigation of the clinical effects of the novel brace. The number of included cases was small and the observation period was short. This may have had a certain impact on the statistical results, and there may be adverse reactions that have not yet emerged. Therefore, the general applicability of the current conclusion awaits further large-sample research. Furthermore, there was no categorization of different types of scoliosis for comparison. The subsequent study will expand the sample size and compare different types of scoliosis patients to further explore and subdivide the efficacy of the novel brace. In this study, the measurement of interfacial pressure was not subdivided according to scoliosis at different locations, but only explored its preliminary characteristics. Subsequent studies should be further subdivided to explore the changing trend of interfacial pressure at different locations. The scales were completed independently by the patients without any external interference. However, due to the small sample size, the subjective feelings of the patients may have a certain impact on the results. Nevertheless, the survey results reveal the main trends. Future research will increase the sample size to minimize this error as much as possible.

The novel brace designed in this study could theoretically improve patients’ wearing compliance, but in actual treatment, patients’ wearing was supervised only through regular follow-up by researchers, which may not accurately reflect the actual wearing situation of patients. Subsequent studies will examine how patients are wearing in real time to identify changes in their compliance.

## Conclusions

For material testing, the bending test and minimum surface temperature of the embedded shape memory alloys met the deformation requirements for the novel spinal orthosis. Because of the weight of shape memory alloys, the new braces may weigh 100–200 g more depending on the patient’s physique and the fixture’s configuration. This may have a slight impact on the comfort of wearing, but currently no patients have reported any significant discomfort during practical application. Since the Ni–Ti shape memory alloy is relatively expensive, the new brace will add a little extra material cost compared to the traditional brace. In clinical trial, the novel brace provided a better three-dimensional correction effect to adolescent idiopathic scoliosis patients and also increased wearing comfort than traditional orthoses, which may improve compliance of patients.

## Materials and methods

### Design of the novel brace

**Fig. 4:**
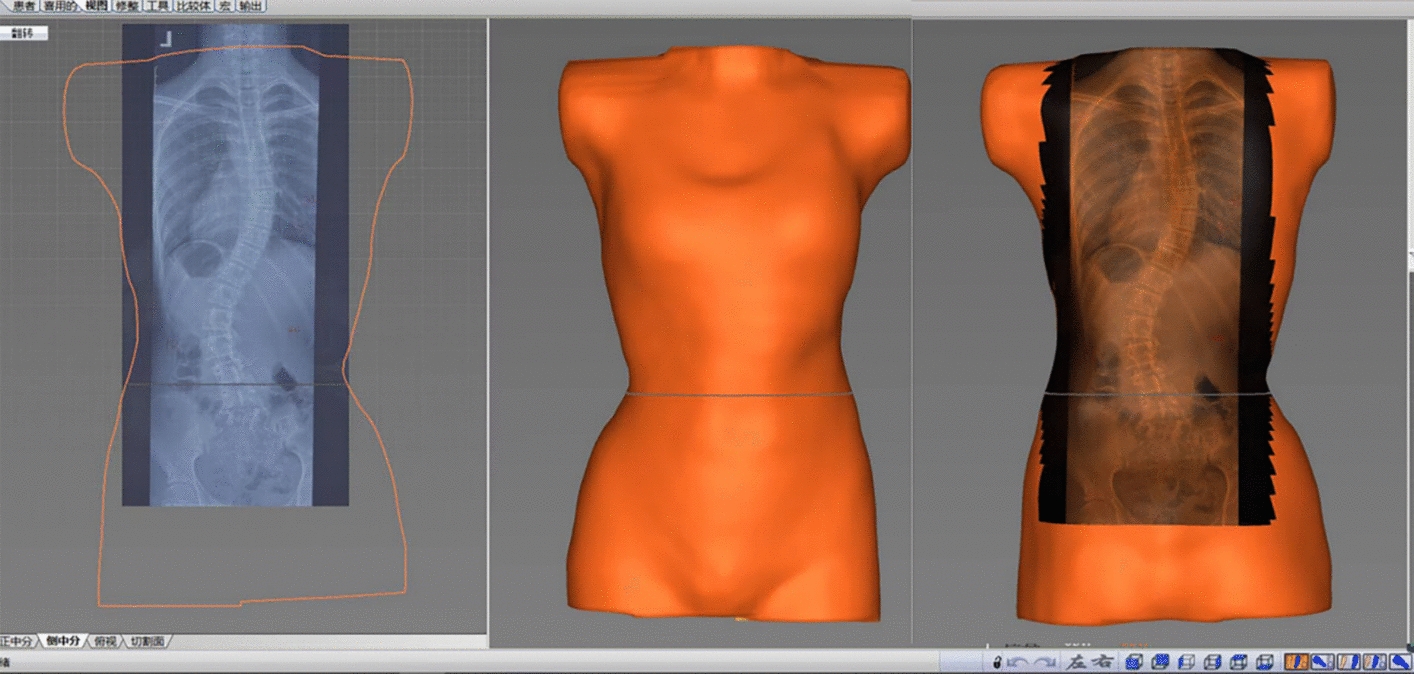
Sketch of the brace design

The novel type of brace was composed of a brace shell and multiple SMA orthopedic pads. The orthopedic pads were placed on the inner side of the brace shell, thus adhering to the patient’s body, and correction was achieved through the deformation of the orthopedic pad. Figure 4 shows the design sketch. The front of the brace had an opening, which could be fastened with adjustable nylon buckles. In theory, the orthopedic pads were placed on the convex side centered on the apical vertebrae, on the concave side centered on the upper end of the vertebrae, and on the concave side centered on the lower end of the vertebrae. In clinical practice, adjustments could be made to the position and quantity of orthopedic pad based on different types of scoliosis in patients and the locations, where corrective force was applied. The outer layer of each orthopedic pad was wrapped with insulating material and an elastic knitted cover, which were fixed at corresponding positions inside of the brace by using Velcro straps.

When the orthosis was used, the orthopedic pads were removed from the freezer compartment of the refrigerator, shaped according to the corresponding position of the patient’s trunk, and fixed to the inner side of the orthosis shell with Velcro, after which the patient wore the orthosis. The nylon buckle was tightened. With the influence of body temperature and environmental temperature, the orthopedic pad underwent a transformation from martensite to austenite and gradually returned to its preset shape. Multiple orthopedic pads acted on the human body at the same time, thus providing patients with continuous three-dimensional corrective force.

### Fabrication of the novel brace

**Fig. 5:**
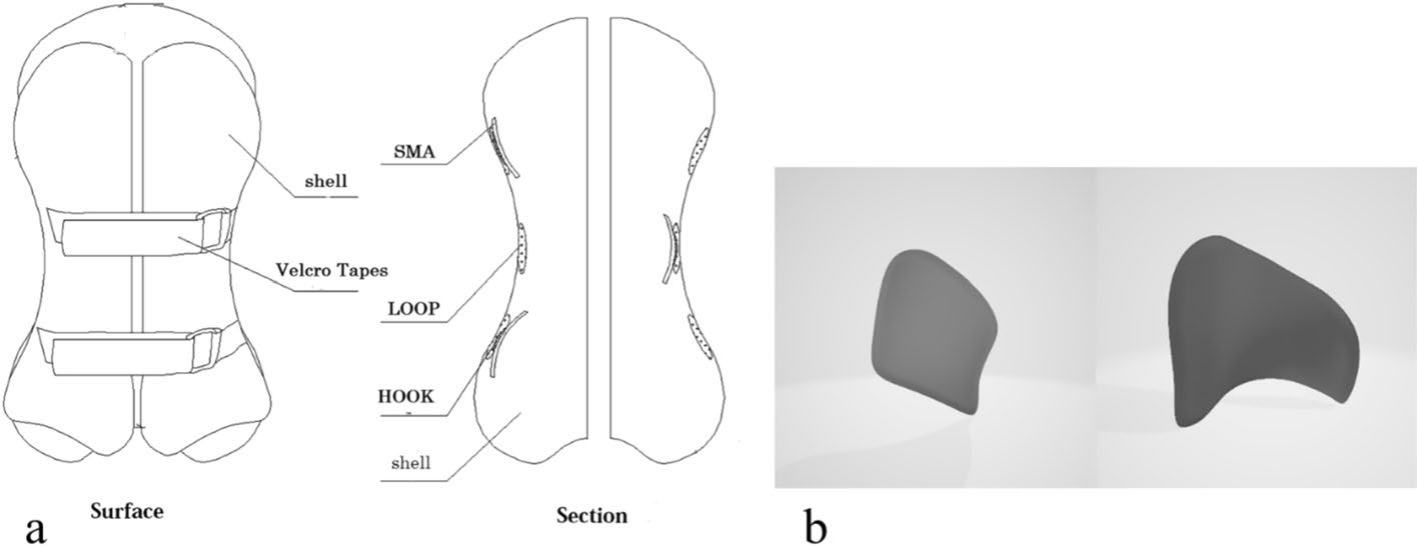
3D fabrication process of the brace (**a**) and orthopedic pad shape (**b**)

The patient’s trunk was scanned by using an optical scanner (PMAX, P3SH202, China), and the image data were imported into a computer. Canfit Design 18.0.4 software was used for shaping to simulate the brace effect on the trunk image model, thus applying corrective force and marking the correction area closer to the posterior–inferior aspect [21] to simultaneously increase both the coronal and sagittal plane corrective forces. Further fine-tuning of the correction area was performed to make its shape as smooth as possible, thus improving patient comfort when wearing it. The correction area was then extracted to form a separate image, which served as the preset shape for the memory alloy orthopedic pads (Fig. 5).

**Fig. 6:**
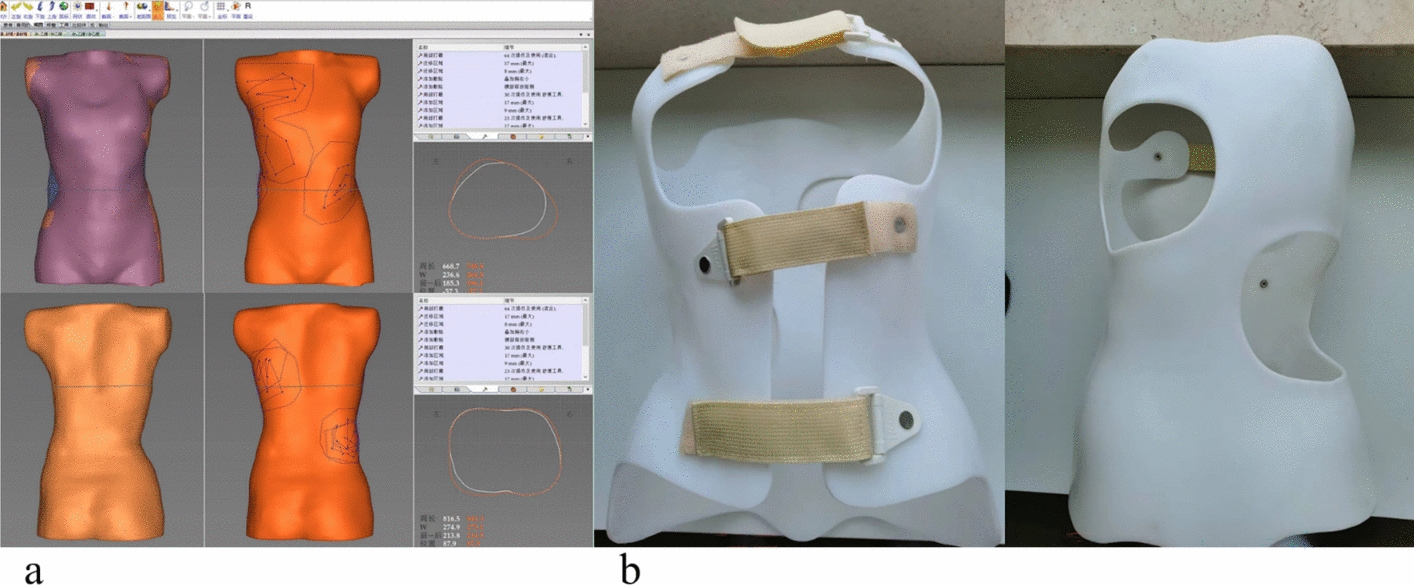
3D fabrication process of the brace (**a**) and brace shells (**b**)

Based on the original trunk model of the patient, the convex lateral space was expanded as a release area to increase the patient’s active correction [21]. The back of the thoracic region was enlarged to conform to the normal physiological curve of the human body. The corrective area released pressure through a lateral window opening, thus preventing excessive compression and avoiding impact on the patient’s growth and development. According to this model, a support shell was made by using a polyethylene board (Fig. 6).

When considering the fact that SMAs, as metal materials, have a certain level of hardness and are relatively difficult to deform in multiple directions, this study chose to use multiple SMA strips arranged in combination to meet different directions of deformation. The orthopedic pads were wrapped in insulation material to reduce the effects of low temperatures on the body while also decreasing the influence of environmental temperature on the orthopedic pad. This helped to delay the deformation time of the orthopedic pad and improve patient comfort during wear.

### Performance test of the brace

#### Bending test

**Fig. 7:**
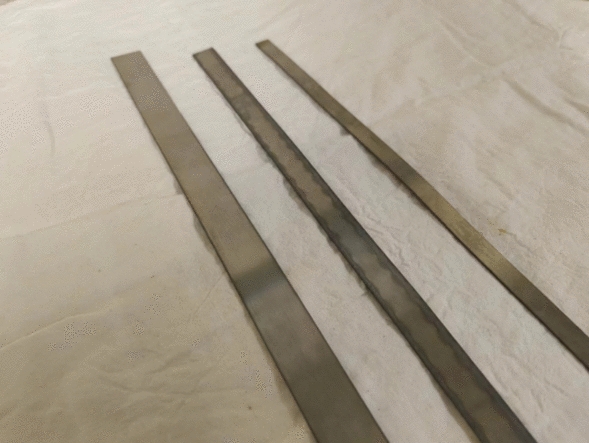
Shape memory alloy samples

The SMA was a Ni‒Ti alloy with widths of 10 mm, 15 mm, and 20 mm and a thickness of 1 mm in this study. The martensitic phase transition temperature was set at 0 °C (refrigerator freezing temperature), and the austenitic phase transition temperature was set at 20 °C (ambient temperature) (Fig. 7).

The SMA was fixed at both ends in the horizontal direction, and a push–pull force gauge (San Liang, SMF, China) was used to push the midpoint of the SMA downward in the vertical direction to induce bending deformation. The pressure values were recorded when the depths were temporarily paused at 5 mm, 10 mm, 15 mm, and 20 mm. SMAs with lengths of 40 cm, 30 cm, 20 cm, and 10 cm were tested at room temperature. Multiple measurements were taken and averaged.

#### Bracing surface temperature test

Patients wearing braces in the ward of Beijing Boai Hospital were selected to measure the temperature of the braces’ surface in different seasons and different states by using a temperature gun (San Liang, FT310, China), and the average value was recorded.

#### Temperature test of the shape memory alloy

The SMA was placed in the freezer and removed, and the temperature was measured at 5 min, 10 min, 15 min and 20 min. After the alloys were allowed to recover to the environmental temperature for 30 min, the recovery temperatures of the SMAs in the laboratory environment (25 ℃) and human body at different times were measured, and the average temperature was recorded.

#### The deformation time of the orthopedic pad

The orthopedic pads were placed in the freezer, removed and shaped after 20 min. The time required for the orthopedic pad to return to the preset shape was recorded.

### Clinical evaluation

#### Inclusion criteria

The included patients were (1) diagnosed with adolescent idiopathic scoliosis; (2) aged 10–15 years; (3) Risser sign ≤ grade 3; (4) mild-to-moderate scoliosis (20° ≤ Cobb angle ≤ 45°); (5) Lenke classification I and V; (6) an apical vertebra under T7; and (7) no other scoliosis treatment received.

#### Exclusion criteria

(1) Patients with nonidiopathic scoliosis; (2) patients with tumors, tuberculosis or other spine-related diseases; (3) patients with severe heart or lung disease; and (4) patients with large areas of skin pressure sores or osteoporosis.

#### How to use the novel brace

The SMA orthopedic pads were placed in the freezer for more than 20 min and then removed and placed at room temperature for 5 min to reduce the surface temperature of the elastic coat and prevent discomfort. The orthopedic pads were placed inside the thermal coat, then fixed on the corresponding position of the inner wall of the brace through Velcro. The patient was asked to wear and tighten the nylon buckles. After tightening the nylon buckle, the bracing can stably adhere to the trunk without significant displacement. Gently pull the edge nylon buckle of the bracing by hand without loosening. There is no abnormal shaking of the support during movement, and the adhesive surface is tightly adhered. The patient has a subjective sense of restraint but no obvious discomfort, and long-term wearing does not cause symptoms, such as pain or numbness.

#### Participants

Ten patients with AIS who were treated at Beijing Boai Hospital from June 2022 to June 2023 were randomly divided into novel brace and control groups. The sample size was determined using the G-power3.1 software. The study protocol was approved by our Institutional Review Board (China Rehabilitation Research Center, Beijing, China) and registered in the Chinese Clinical Trials Registry (No. ChiCTR2400085984, 21/06/2024). All experiments were performed in accordance with the Declaration of Helsinki. All of the participants and their families were informed in detail about the content of the study and signed informed consent before enrollment in the study.

#### Methods of clinical evaluation

**Fig. 8:**
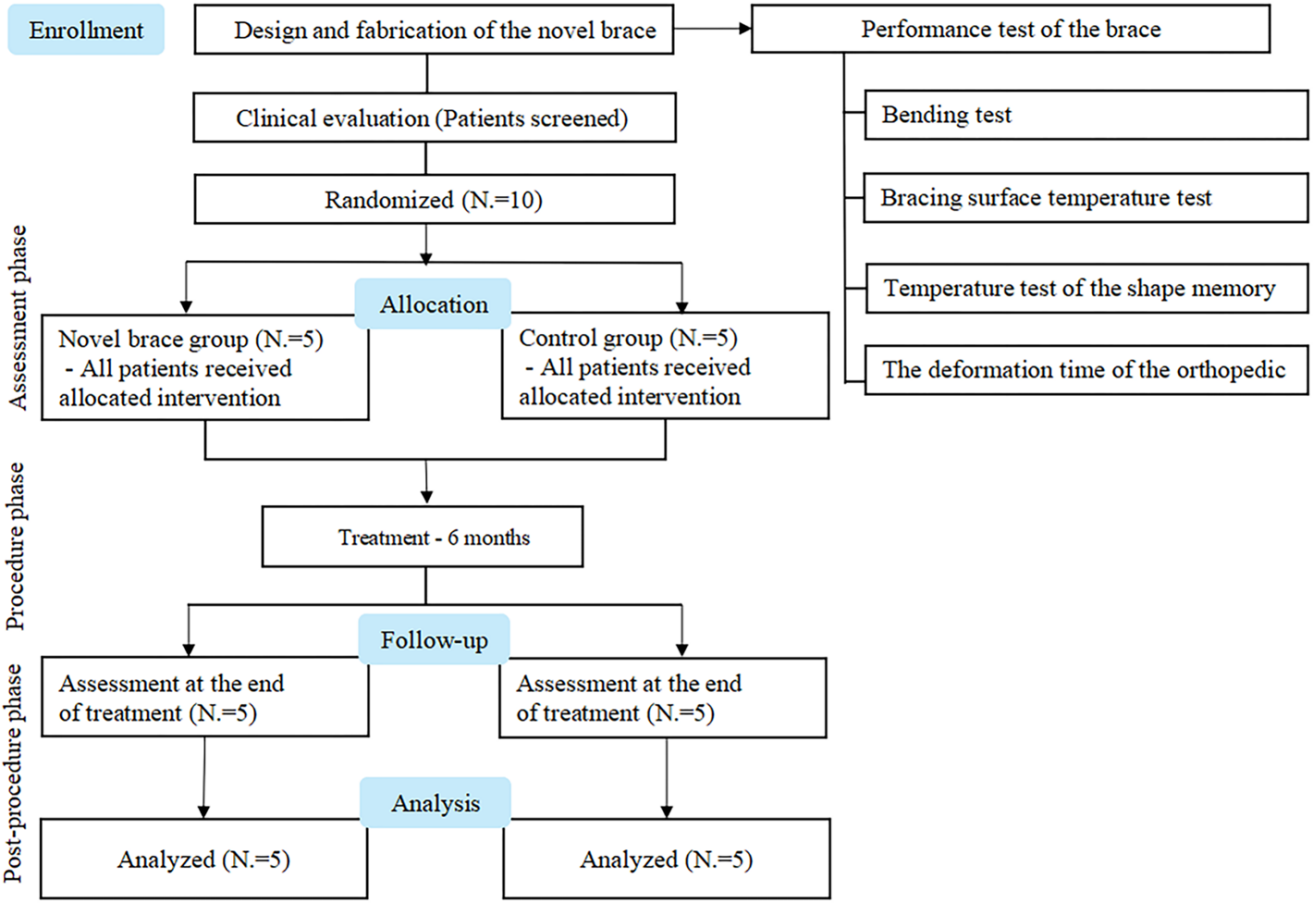
Flow chart of randomization and phases of the study

This study is a prospective randomized controlled trial. Patients who met the inclusion criteria were randomly assigned to either the novel brace group or the traditional brace control group by using random allocation software. Generate a random sequence of numbers from 1 to 10 using Excel. The numbers located at odd positions are classified as the experimental group, while those at even positions are assigned to the control group. Record the complete random sequence, seal it and archive it. After patients are enrolled, they are grouped according to their order of enrollment. Prior to treatment, the patients underwent evaluation of their standing spine via anteroposterior and lateral X-ray images by a professional spinal surgeon. The brace pad was designed by the authors of this study. The shaping and fabrication of the braces were completed by experienced orthotists, with the production and processing of SMAs being performed at the School of Materials Science and Engineering, University of Science and Technology Beijing. The correction methods of both sets of braces are based on the principle of three-dimensional correction. Traditional braces rely on linear compression, with smaller area and more concentrated force points. The new brace uses an SMA pad for dynamic three-dimensional correction instead of simply using diagonal compression of the brace shell. Patients tried on the braces to confirm initial comfort and to make necessary adjustments if discomfort was present. Subsequently, patients wore the braces for more than 20 h per day. All of the subjects underwent a follow-up examination after wearing the orthoses for 6 months [22], during which they had new anteroposterior and lateral spine X-rays performed after removing the orthoses for 12 h, followed by the measurement and recording of relevant indicators. Throughout treatment, researchers regularly followed up with patients through telephone to assess any discomfort and supervise compliance with wearing instructions. The complete experimental procedure is presented in Table 10, which is in accordance with the *TIDieR* template for intervention description and replication. Allocation was concealed and communicated by telephone to investigators (by a third person). Figure 8 shows the flowchart of patient randomization and phases of the study.

**Table 10:** TIDieR reporting checklist

Brief name	A novel spinal orthosis with embedded shape memory alloy for treatment of adolescent idiopathic scoliosis
WHY: rationale	Braces are an effective intervention method for treating mild-to-moderate adolescent idiopathic scoliosis. The novel orthosis developed in this study can enhance the three-dimensional correction effect and improve patient compliance
WHAT: materials	The experimental group received a 6-month intervention with the novel orthosis, while the control group received a 6-month intervention with the traditional rigid orthosis. The design and production process of the new orthosis, as well as the specific intervention details of the participants, can be found on page 11 of the original text [Page 11, Line 22]
WHAT: procedures	Customized orthoses are made based on the results of patients' imaging examinations and optical scanning data. Patients and their families will receive training on how to use the orthoses correctly. Under the supervision of clinical doctors and guardians, patients will undergo a 6-month orthotic treatment plan, wearing the orthoses for more than 20 h per day, and spending the rest of the time on personal hygiene and necessary activities. After 6 months, re-imaging examinations will be conducted to evaluate the effectiveness of the intervention measures. Clinical doctors will maintain regular contact with patients and their families, record the compliance with wearing requirements, and ensure that patients adhere to the treatment
WHO provided	The graduate student responsible for patient recruitment and assessment received training under the guidance of a spinal surgeon. Each assessment was jointly conducted by a spinal surgeon with over 10 years of experience. The orthotist has over 5 years of experience in the manufacturing of orthoses, and the design and production of shape memory alloy materials were supervised by experts in the field. The principal investigator had continuous and in-depth discussions with all participants in the research project and maintained regular communication regarding progress and results
HOW: mode of delivery	The patient was evaluated at the beginning of the study, who met the inclusion criteria received custom orthoses within 2 weeks. The researchers conduct weekly online consultations with the patients and their families to monitor the intervention measures. After 6 months of treatment, an imaging examination will be conducted for review to assess the treatment outcome
WHERE: where the intervention occurred	Data assessment and collection are conducted within the hospital. The intervention sites vary depending on the patient's destination, mainly at home or at school. Researchers maintain regular online communication with the patients
WHEN and how much	The treatment period is 6 months. The patient needs to wear the orthosis for more than 20 h every day
Taloring	Due to the changes in the patient's condition and developmental stage, each orthosis is custom-made according to the individual specifications. The design and manufacturing are tailored to the patient's height, weight, and the angle of spinal deformity, and these processes are completed after the first enrollment. A follow-up assessment is conducted 6 months later, and the patient is required to return to the hospital for a re-evaluation
Modifications	Due to differences in their living locations, patients may not always be able to return to the hospital on time. Three patients underwent imaging follow-up examinations at the local hospital after the treatment, and then returned to our hospital within the following few days to submit the results for evaluation
HOW well: planned	The patient's compliance was evaluated through online follow-up, based on the self-reports of the patients and their families regarding the use of orthoses
HOW well: actual	During the communication with the patients and their families, we ascertained that all patients had completed the prescribed course of treatment. However, based on the assessment of outcome measures, we believe the patients' accounts were not entirely accurate

TIDieR Report Checklist from www.equator-network.org

#### Assessments

The coronal Cobb angle, thoracic kyphosis angle, lumbar lordosis angle and apical rotation angle were measured before and after treatment to evaluate the progression and improvement of scoliosis. Due to the limited sample size, Cobb angle measurements for patients were obtained solely from the primary curve. The AVR was measured using the Nash–Moe method. The grade is based on the displacement of the apical vertebral pedicle; the greater the displacement, the higher the grade. Imaging examinations were performed using an X-ray machine (SHIMADZU, RADspeed Pro 50, China). A film pressure transducer (CHENG TEC, MY2802, China) was used to test the interface pressure on the skin of the main correction area. The thin-film pressure sensor was positioned between the brace’s pressure pad and the patient’s trunk. The pressure was measured when the upper body was braced and after the orthosis was deformed in the novel brace group (with the deformation of the orthosis, the value displayed by the pressure tester increased; when the value was stable and no significant change occurred, the value was recorded as the pressure after deformation). The pressure of the control group after wearing the brace was measured. All data were averaged after multiple measurements. The Scoliosis Research Society-22 (SRS-22) scale and the Quebec Assistive Technology User Satisfaction scale were used to evaluate the patients’ subjective assessment before and after treatment.

### Statistical analysis

All of the data were analyzed with SPSS Statistics 23. Continuous data are expressed as the mean ± standard deviation (^−^X ± s). Normality tests were conducted for each group, and independent sample *t* tests were used for between-group comparisons if the data followed a normal distribution. Paired sample *t* tests were used for within-group comparisons, whereas non-normally distributed data were analyzed by using the Mann‒Whitney *U* test. Frequency was used to represent unordered categorical data, and between-group comparisons were made by using the chi-square test. The Mann–Whitney *U* test was used for between-group comparisons of ordinal data. *P* < 0.05 was considered statistical significance.

## Data Availability

The data associated with this paper are not publicly available but are available from the corresponding author on reasonable request.
